# Parental control and depressive symptoms in college freshmen with myopia: the mediating role of vision-related quality of life

**DOI:** 10.3389/fpsyt.2025.1642896

**Published:** 2025-12-26

**Authors:** Gang Liang, Xing-Xuan Dong, Dan-Lin Li, Min-Xin Liu, Zhi-Jian Yin, Yue-Zu Li, Tianyang Zhang, Chen-Wei Pan

**Affiliations:** 1 Department of Ophthalmology, the Affiliated Hospital of Yunnan University, Kunming, China; 2 School of Public Health, Suzhou Medical College of Soochow University, Suzhou, China; 3 Department of Ophthalmology, the First Affiliated Hospital of Dali University, Dali, China; 4 Graduate School of Interdisciplinary Science and Engineering in Health Systems, Okayama University, Okayama, Japan; 5 Research Center for Psychology and Behavioral Sciences, Soochow University, Suzhou, China

**Keywords:** depressive symptoms, parental control, quality of life, myopia, college freshmen

## Abstract

**Objectives:**

Myopia is considered to be associated with depressive symptoms. Perceived parental control plays a critical role in children and adolescents’ mental health. The present study aimed to examine the role of myopia in depressive symptoms and whether parental control contributes to depressive symptoms via vision-related quality of life (QoL) in myopic students.

**Methods:**

This study investigated the mediating role of vision-related QoL between parental control and depressive symptoms among Chinese children and adolescents with myopia. A total of 2014 college freshmen in China were included. Correlation analysis and mediation analysis were conducted. Mediation models were used to assess the potential mechanism of vision-related QoL in the association between parental control and depressive symptoms.

**Results:**

In our sample, participants with myopia were less likely to develop depressive symptoms than those without myopia (P = 0.01). Subsequently, analysis within the myopic group revealed that vision-related QoL significantly mediated the relationship between parental control and depressive symptoms, accounting for 15.69% (maternal control) and 18.86% (paternal control) of the total effect.

**Conclusions:**

This study highlights the important role of myopia for depressive symptoms in college freshmen and demonstrates that vision-related QoL significant mediates the relationship between parental control and depressive symptoms.

## Introduction

The prevalence of myopia has markedly increased during recent decades, especially in East Asia (1). The prevalence of myopia in Shanghai has been reported as 95.5% (2). A university-based study conducted between 2016 and 2021 found that myopia among mainland Chinese college freshmen in Tianjin remained both stable and elevated, ranging from 91.8% to 94.9% (3). In Taiwan, a longitudinal study documented a marked increase from 91.3% (1988) to 95.9% (2005) among college freshmen (4). Beyond its impact on vision, myopia imposes tremendous economic costs and elevates lifelong risks of secondary sequelae, including glaucoma and cataracts (5), cementing its status as a critical public health priority. Myopia has become the main risk factor impeding the development of children and adolescents, and is considered a major public health concern.

Myopia has a significant impact on mental health, except for its well-known adverse consequences. Depression and depressive symptoms, as common psychological illnesses, have been proven to lead to suicide (6) and disability (7) in previous studies. The peak prevalence of depression generally occurs in the second and third decades of life, which indicates that children and adolescents are susceptible groups for depression (8). Although the prevalence of clinical depression is low, depression symptoms are relatively common. Approximately 3.2% of US children have depression, while 17.3% of Singapore children suffer from depressive symptoms (9). Previous studies support that psychological problems are associated with poor emotional regulation (10). However, the relationship between myopia and depression remains complex and inconsistently reported. While some studies suggest a significantly higher risk of depression among students aged 13–17 years with myopia than in those without (11), and a systematic review showed that children and adolescents with myopia report significantly higher depression scores than their emmetropic peers (standardized mean difference = 0.58) (12), other investigations have found no significant association (13, 14). This inconsistency suggests that the link between myopia and depression may vary across populations and study designs, highlighting the need for further investigation in specific demographic groups. It should be noted that children and adolescents are a high-risk group for depression and depressive symptoms, and myopia may impose an additional psychological burden. However, its onset can also trigger adaptive health behaviours. Latent-class analysis revealed that 73.7% of myopic students belong to a “health-behaviour” subtype (15). These behaviours, including increased physical activity and improved sleep hygiene, may exert an independent antidepressant effect (16).

Myopia not only affects children and adolescents but also impacts and is reciprocally influenced by their family, especially immediate family members. Parents of children with myopia report markedly higher psychological distress than parents of non-myopic children; this distress is driven mainly by persistent concerns about visual prognosis and the long-term risk of sight-threatening complications (17). To mitigate these anxieties and protect ocular health, parents may prefer to intensify behavioural-control strategies (17). Parental control, in this context, includes actions such as regulating screen time, restricting physical activities for fear of injury, and mandating the consistent use of corrective lenses. These behaviours are measured using the Parental Bonding Instrument (PBI) in our study, which assesses the levels of control and affection in parental interactions. While moderate parental involvement in vision-protective practices, such as encouraging outdoor activity, decreases the risk of myopia (18), excessive control, particularly psychological control, can independently impair children’s emotional regulation and overall mental well-being.

Previous studies have shown a significant association between parenting styles and quality of life (QoL), indicating that families play a critical role in increasing children and adolescents’ QoL (19). Moreover, a cross-sectional survey conducted in China suggested that parenting styles were significantly associated with QoL (20). Specifically in the context of myopia, vision-related QoL provides a multi-dimensional framework to capture the unique challenges faced by adolescents with visual impairment, encompassing physical function, emotional function, vision function and body performance, and social activity domains. Parenting styles influence children’s appraisals of bodily competence through physical self-esteem, which may contribute to depressive symptoms (21, 22). Overcontrolling parents, who prohibit “high-risk” physical activities or prioritize academic work over exercise, may inadvertently increase their children’s vulnerability to depression. Psychological control directly undermines university students’ self-worth and emotion-regulation capacity, fostering anxiety and, consequently, depressive symptoms (23). Excessive parental intervention, such as mandating specific spectacles, accentuates the functional limitations and helplessness associated with visual impairment. Previous evidence has indicated that visual impairment and its functional restrictions are independent risk factors for depression (24, 25). Social support is considered as a protective factor against depression (25, 26). However, when parental control extends to the social domain, constraining friendships and limiting interaction time/space, both the quality and quantity of social support may diminish.

It has been reported that authoritative parenting may be beneficial to the psychological development of children and adolescents, meaning that parenting styles can affect the psychological well-being of offspring (27). Similarly, a cohort study suggested that the mental health of children and adolescents is greatly affected by parenting styles (28). Furthermore, the risk of depression and suicidal ideation may be increased by parental control and decreased by parental care (28). Negative parenting styles, such as apathetic and controlling parenting, significantly increase the risk of depression, and the higher the levels of parental control are, the worse the depressive symptoms of adolescents (29) will become. In addition, previous studies found that QoL may decline due to the presence of depression (30–32) and improve after its remission (32).

College freshmen are a stressful group in the Chinese context because they are experiencing a series of dramatic transitions in their lives (33). Specifically, students in the first year of college are facing the sadness of parting with friends and family, heavy academic pressure and fear of being in a completely unfamiliar environment (34). Therefore, stress levels escalate amidst this life upheaval, increasing their susceptibility to depression and anxiety (35). Considering the high prevalence of myopia and a variety of adverse consequences caused by depression, it is imperative to understand the role of parents in the development of emotional adjustment in children and adolescents. In particular, frequent psychological distress associated with myopia in children and adolescents may hinder the completion of important developmental tasks to varying degrees.

Although current studies have uncovered the association between myopia and mental health, and have provided preliminary evidence for the associations among parental control, vision-related QoL, and depressive symptoms, several critical knowledge gaps remain. First, first-year university students, who confront simultaneous environmental, academic and identity transitions, remain under-studied. Second, the mediating pathways linking parental control to depressive symptoms via vision-related life have not been examined within myopic samples, and the cultural specificity of “overprotection” in Chinese families remains largely unexplored. These controlling behaviors (e.g., prioritizing academics over exercise, restricting social interactions, mandating specific vision correction methods) may be particularly salient or manifest in culturally specific ways within Chinese families, where academic achievement and safety concerns are often paramount. In this study we aim to (a) investigate the differences between college freshmen with myopia and without, (b) test the effects of parental control on depressive symptoms in college freshmen with myopia, and (c) examine whether the relationship between parental control and depressive symptoms is mediated by vision-related QoL in college freshmen with myopia. Overall, we tested the following two major hypotheses:

Hypothesis 1. The status of depressive symptoms differs between myopic and non-myopic participants.

Hypothesis 2. Parental control may contribute to depressive symptoms via vision-related QoL.

## Methods

### Study design and participants

The Dali University Students Eye Health Study was was conducted in 2021 and a school-based study conducted in Dali among college freshmen in Yunnan Province, located in the south-western part of China. According to official records, all freshmen (*n* = 2698) at Dali University were invited to participate in this study. Participants who were older than 26 years, submitted invalid questionnaires, or had eye diseases (i.e., keratoconus, acute infection) were excluded. Finally, a total of 2014 individuals completed physical examinations, underwent eye examinations including IOL Master and optical coherence tomography (OCT) and provided blood samples, and were thus included in this analysis. The characteristics of age, gender, ethnicity and parental myopia revealed no statistically significant differences between the included and excluded students.

### Instruments

A standardized questionnaire was used to collect information on demographic variables (i.e., gender, age, and ethnicity) and lifestyle factors (i.e., history of smoking and drinking). Home address was categorized as “city”, “town”, or “county”. Ethnicity was classified as “Han” and “non-Han”. Family type was categorized as “two-parent” or “single-parent/no-parent”. Family socioeconomic status was classified as “good”, “moderate”, or “poor”. Body mass index (BMI) was calculated as weight divided by height squared.

Refractive error (sphere, cylinder, and axis) was measured using an autorefractor (Canon Inc. Ltd., Tochigiken, Japan) operated by optometrists or trained technicians. Myopia was recorded in increments of 0.25 Diopters. The first five valid readings were used and averaged using vector methods to give a single estimate of refractive error. All five readings were required to be at most 0.50 diopters (D) apart in both the spherical and cylinder components. The spherical equivalent (SE) was defined as the sphere plus half cylinder. Myopia was defined as an SE of less than -0.5 D in the current study (24).

The PBI is a widely used self-report measure assessing perceived parenting styles during the first 16 years across three dimensions: parental care, autonomy encouragement, and control (36, 37). Both maternal (PBI-M) and paternal (PBI-F) versions were administered, each containing 23 items rated on a 4-point Likert scale (0 = “very inconsistent” to 3 = “very consistent”), with higher scores indicating stronger parental behaviors. We focused on its parental control subscale, which evaluates intrusive and overprotective parental behaviors that restrict a child’s independence and psychological autonomy. This subscale comprises 6 items (e.g., restricting activities, imposing excessive control). This subscale has demonstrated good reliability and validity in Chinese college freshmen (38). Cronbach’s alpha for the control subscale was 0.536 (PBI-M) and 0.595 (PBI-F), which is acceptable given the limited number of items and the multi-dimensional nature of parental control.

A vision-related QoL scale was used to assess the QoL of college freshmen with myopia (39). The 22-item scale comprises four subscales: Physical Function (interference with daily physiological activities due to visual defects), Emotional Function (emotional changes caused by vision defects), Vision Function and Bodily Performance (limitations in vision and physical function due to vision defects), and Social Activity (restrictions in social activities due to vision defects). Items are rated on a 5-point scale (1 = “always” to 5 = “never”), with higher scores indicating better QoL. Subscale score ranges are 6–30 for Physical Function, 9–45 for Emotional Function, 5–25 for Vision Function and Bodily Performance, and 2–10 for Social Activity; the total score ranges from 22-110.

The Self-Rating Depression Scale (SDS) is a 20-item self-report questionnaire assessing depressive symptoms over the preceding week (40–42). Items are rated on a 4-point Likert scale (1 = “a little of the time” to 4 = “most of the time”), yielding a total score range of 20-80. Cronbach’s alpha was 0.857.

### Data management and statistical analysis

Data are expressed as the mean ± standard deviation (SD) for continuous variables and as frequency or percentage for categorical variables. Characteristics of participants with myopia versus those without myopia were compared using Student’s t test or the chi-square test. Pearson’s bivariate correlation analysis was used to evaluate associations between study variables.

To determine whether vision-related QoL mediates the relationship between parental control and depressive symptoms, a mediation analysis was performed using PROCESS (version 3.5 by Andrew F. Hayes) with Model 4. The mediation models were adjusted for age, gender, BMI, ethnicity, alcohol use, smoking status, family socioeconomic status, home address, family type, and only-child status. Robustness was evaluated by calculating *E* values for the total effect to quantify the potential impact of unmeasured confounders (43, 44). *Cohen’s f^2^* was calculated to describe the magnitude of the association between parental control and depressive symptoms (45). The significance of the mediation effect was tested using 5,000 bootstrap resamples to construct a 95% confidence interval (*CI*). A mediating effect was considered significant if the 95% *CI* excluded zero. Statistical power for each pathway within the mediation model was estimated using the wp.mediation function (WebPower, R). Separate analyses were run for each vision-related QoL subscale: physical function, emotional function, vision function and bodily performance, and social activity, with α set at 0.05 (two-tailed). All statistical analyses were performed using SPSS version 25.0 and R version 4.3.1. Two-sided *P* values < 0.05 were considered statistically significant.

## Results

Among the 2,698 freshmen, 2,014 provided complete data, yielding an effective response rate of 74.6%. **Table 1** summarizes the characteristics of the entire sample. **Table 2** presents descriptive statistics and bivariate correlations among the main study variables, which provide the basis for the subsequent mediation analysis. Maternal and paternal control were positively associated with depressive symptoms in both groups (myopic: *r* = 0.31 and 0.33; non-myopic: *r* = 0.21 and 0.28, all *P* < 0.05). Among myopic participants, paternal control was negatively correlated with physical function (*r* = -0.11), emotional function (*r* = -0.21), visual function and body performance (*r* = -0.17), and social activity (*r* = -0.05). Maternal control was significantly correlated with physical function (*r* = -0.08), emotional function (*r* = -0.21) and visual function and body performance (*r* = -0.18) in myopic participants. Except for social activity, parental control was significantly related to all study variables in non-myopic participants (*P* < 0.05).

**Table 1:** Sociodemographic and measures characteristics.

Characteristics	Students with myopia (n=1848)	Students without myopia (n=166)	*P* value
Gender, n(%)			
Male	554 (87.00)	83 (13.00)	< 0.001
Female	1294 (94.00)	83 (6.00)	
Age (years)	19.00 (0.93)	18.99 (0.89)	0.91
Body mass index (kg/m^2^)	20.82 (3.37)	20.60 (2.86)	0.35
Ethnicity			
Han	1391 (92.06)	120 (7.94)	0.40
Non-Han	457 (90.85)	46 (9.15)	
Alcohol status, n(%)			
Yes	311 (87.11)	46 (12.89)	< 0.001
No	1537 (92.76)	120 (7.24)	
Smoke status, n(%)			
Yes	118 (82.52)	25 (17.48)	< 0.001
No	1730 (92.46)	141 (7.54)	
Family condition			
Good	751 (91.47)	70 (8.53)	0.34
Moderate	1043 (92.22)	88 (7.78)	
Poor	54 (87.10)	8 (12.90)	
Home address			
City	275 (90.46)	29 (9.54)	0.29
Town	364 (93.57)	25 (6.43)	
County	1209 (91.52)	112 (8.48)	
Family type			
Two-parent	1659 (91.86)	147 (8.14)	0.62
Single-parent or no-parent	189 (90.87)	19 (9.13)	
Only child			
Yes	315 (88.73)	40 (11.27)	0.02
No	1533 (92.41)	126 (7.59)	
Depressive symptoms			
Yes	913 (82.18)	99 (17.82)	0.01
No	935 (93.31)	67 (6.69)	
Maternal control	6.32 (2.76)	6.84 (2.82)	0.02
Paternal control	6.15 (2.92)	6.65 (3.07)	0.03

**Table 2:** Descriptive statistics of and linear correlations among the study variables (n = 2014).

	Mean	SD	1.	2.	3.	4.	5.	6.	7.
Myopia									
1. Depressive symptoms	41.26	8.45	1.00						
2. Physical function	20.13	4.62	-0.11^***^	1.00					
3. Emotional function	34.75	5.68	-0.26^***^	0.61^***^	1.00				
4. Vision function and body performance	20.43	4.24	-0.21^***^	0.50^***^	0.68^***^	1.00			
5. Social activity	5.76	2.60	-0.23^***^	-0.18^***^	-0.11^***^	-0.13^***^	1.00		
6. Maternal control	6.32	2.76	0.31^***^	-0.08^***^	-0.21^***^	-0.18^***^	0.01	1.00	
7. Paternal control	6.15	2.92	0.33^***^	-0.11^***^	-0.21^***^	-0.17^***^	-0.05^*^	0.62^***^	1.00
Without myopia									
1. Depressive symptoms	42.55	8.41	1.00						
2. Physical function	24.30	4.81	-0.14	1.00					
3. Emotional function	39.61	5.71	-0.31^***^	0.65^***^	1.00				
4. Vision function and body performance	23.21	3.38	-0.25^***^	0.67^***^	0.87^***^	1.00			
5. Social activity	5.75	3.38	-0.31^***^	-0.18^*^	0.10	-0.03	1.00		
6. Maternal control	6.84	2.82	0.21^*^	-0.19^*^	-0.20^*^	-0.20^*^	-0.10	1.00	
7. Paternal control	6.65	3.07	0.28^***^	-0.20^*^	-0.24^*^	-0.23^*^	-0.06	0.64^***^	1.00

*P <.05; ***P <.001.

Mediation analysis was conducted to examine whether vision-related QoL mediated the association between parental control and depressive symptoms. As shown in **Table 3**, vision-related QoL significantly mediated these associations. The mediating effect of maternal and paternal control was 0.15 (15.69%) and 0.18 (18.86%), respectively. Standard indirect effects of maternal and paternal control on depressive symptoms were reported in **Table 3** (maternal: 0.05 ± 0.01; paternal: 0.06 ± 0.01). Without vision-related QoL, the total effects of maternal (*β* = 0.93, *P* <.001) and paternal (*β* = 0.94, *P* <.001) control on depressive symptoms were significant. *Cohen’s f²* values for the total-effect model were 0.13 (maternal control) and 0.15 (paternal control), the latter meeting and the former approximating the conventional medium-effect threshold of 0.15, indicating a moderate effect size between parental control and depressive symptoms. The *E* values for the associations between maternal control, paternal control, and depressive symptoms were all 1.45 (**Supplementary Figure 1**). An *E*-value of 1.45 indicates that an unmeasured confounder would need to be associated with both parental control and depressive symptoms by a risk ratio of at least 1.45 to fully explain away the observed association, which is considered moderately strong, increasing confidence in the results. However, no such confounder was observed in our study. Therefore, we proposed that any omitted confounder was unlikely to explain the observed findings, attesting to the robustness of the results.

**Table 3:** The mediating effect of vision-related quality of life with depressive symptoms as the dependent variable (n = 1848).

Model effect	Effect size	SE	95% CI		Proportion of effect
			LCI	UCI	
Maternal control					
Total effect	0.93	0.07	**0.79**	**1.06**	100.00%
Direct effect	0.78	0.07	**0.65**	**0.91**	84.31%
Indirect effect	0.15	0.03	**0.09**	**0.20**	15.69%
Model 1	-0.01	0.01	-0.03	0.01	
Model 2	0.14	0.03	**0.09**	**0.19**	
Model 3	0.04	0.02	**0.002**	**0.07**	
Model 4	-0.02	0.02	-0.05	0.02	
Standard indirect effect	0.05	0.01	**0.03**	**0.07**	
Model 1	-0.003	0.003	-0.01	0.002	
Model 2	0.04	0.01	**0.03**	**0.06**	
Model 3	0.01	0.01	**0.001**	**0.02**	
Model 4	-0.01	0.01	-0.02	0.01	
Paternal control					
Total effect	0.94	0.06	**0.81**	**1.06**	100.00%
Direct effect	0.76	0.06	**0.64**	**0.88**	81.14%
Indirect effect	0.18	0.03	**0.13**	**0.23**	18.86%
Model 1	-0.02	0.01	-0.04	0.01	
Model 2	0.13	0.03	**0.08**	**0.18**	
Model 3	0.03	0.02	**0.01**	**0.07**	
Model 4	0.03	0.02	-0.002	0.06	
Standard indirect effect	0.06	0.01	**0.05**	**0.08**	
Model 1	-0.01	0.004	-0.01	0.002	
Model 2	0.04	0.01	**0.03**	**0.06**	
Model 3	0.01	0.01	**0.002**	**0.02**	
Model 4	0.01	0.01	-0.001	0.02	

Models 1 to 4 represent the mediating pathways mediated by Physical Function, Emotional Function, Vision Function and Body Performance, and Social Activity, respectively. Bold font indicates a significant model.

**Figure 1:**
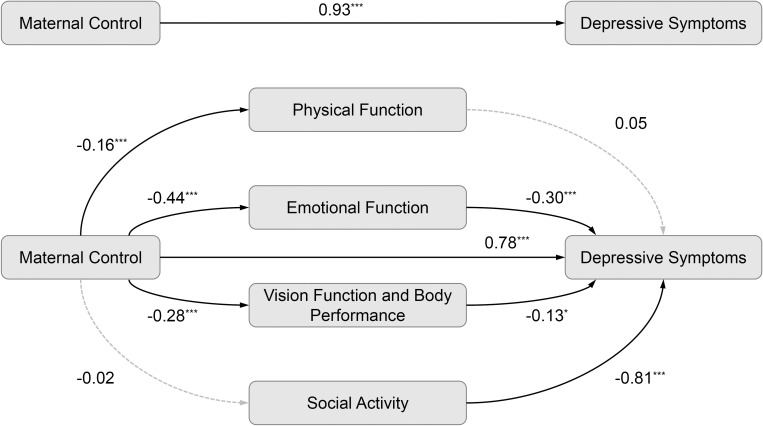
The mediation analysis with maternal control among college freshmen with myopia (*n* = 1848). *P <.05; ***P <.001; solid lines indicate significant paths; dashed lines indicate non-significant paths.

**Figure 2:**
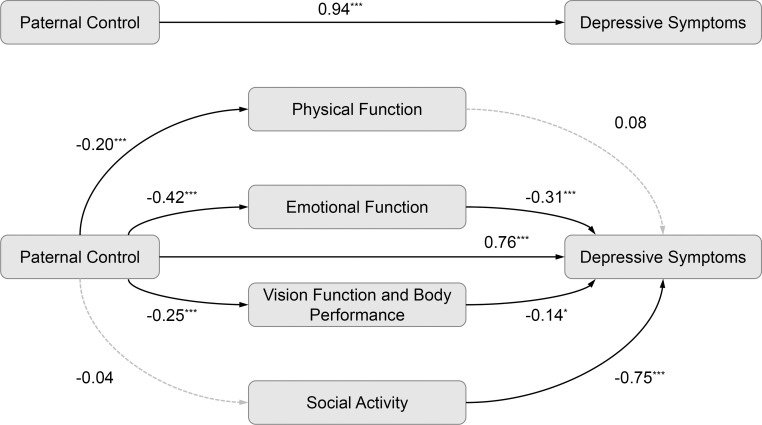
The mediation analysis with paternal control among college freshmen with myopia (*n* = 1848). *P <.05; ***P <.001; solid lines indicate significant paths; dashed lines indicate non-significant paths.

**Figure 1** shows that higher levels of maternal control were associated with poorer physical function (*β* = -0.16, *P* <.001), emotional function (*β* = -0.44, *P* <.001), and vision function and body performance (*β* = -0.28, *P* <.001). Emotional function (*β* = -0.30, *P* <.001) and vision function and body performance (*β* = -0.13, *P* = .03) significantly associated with depressive symptoms. Similarly, **Figure 2** indicates that higher paternal control was linked to lower scores on physical function (*β* = -0.20, *P* <.001), emotional function (*β* = -0.42, *P* <.001), and vision function and body performance (*β* = -0.25, *P* <.001). Emotional function (*β* = -0.31, *P* <.001) and vision function and body performance (*β* = -0.14, *P* = .02) significantly linked to depressive symptoms.

**Table 4** suggests the results of the bootstrap test for mediation effects based on 5,000 resamples, which are consistent with the previously specified mediation models. In the association between maternal control and depressive symptoms, the indirect effect via emotional function was significant (*β* = -0.44, 95% *CI* = -0.56 to -0.34), as was the indirect effect via vision function and bodily performance (*β* = -0.28, 95% *CI* = -0.36 to -0.21). Physical function and social activity achieved statistical significance only along isolated segments, thereby failing to establish a complete mediation chain. Parallel findings were observed for paternal control, with emotional function (*β* = -0.42, 95% *CI* = -0.53 to -0.32) and vision function and bodily performance (*β* = -0.25, 95% *CI* = -0.32 to -0.17) emerging as robust mediators, whereas physical functioning and social activity remained significant solely in single segments. The *post-hoc* power analysis indicated that the pathway via emotional function achieved near-maximal power (power > 0.99). The pathway through vision function and bodily performance reached a moderate level (power = 0.61), whereas the pathways via physical function (power = 0.232) and social activity (power = 0.276) exhibited comparatively low power.

**Table 4:** Results of bootstrap test for mediating effects (5000 resamples).

Mediation models	Effect size	Boot mean	Boot SE	Bootstrap 95% CI	
				Boot LCI	Boot UCI
Model 1					
Maternal Control→					
Physical Function	-0.16	-0.17	0.05	**-0.25**	**-0.08**
Emotional Function	-0.44	-0.45	0.06	**-0.56**	**-0.34**
Vision Function and Body Performance	-0.28	-0.28	0.04	**-0.36**	**-0.21**
Social Activity	0.02	0.02	0.02	-0.02	0.07
Depressive Symptoms	0.78	0.78	0.07	**0.65**	**0.91**
Depressive Symptoms←					
Physical Function	0.05	0.05	0.05	-0.05	0.14
Emotional Function	-0.30	-0.30	0.05	**-0.40**	**-0.21**
Vision Function and Body Performance	-0.13	-0.13	0.06	**-0.24**	**-0.01**
Social Activity	-0.81	-0.81	0.07	**-0.95**	**-0.66**
Model 2					
Paternal Control→					
Physical Function	-0.20	-0.20	0.04	**-0.29**	**-0.11**
Emotional Function	-0.42	-0.42	0.05	**-0.53**	**-0.32**
Vision Function and Body Performance	-0.25	-0.25	0.04	**-0.32**	**-0.17**
Social Activity	-0.04	-0.04	0.02	-0.08	0.002
Depressive Symptoms	0.76	0.76	0.06	**0.64**	**0.88**
Depressive Symptoms←					
Physical Function	0.08	0.08	0.05	-0.03	0.18
Emotional Function	-0.31	-0.31	0.05	**-0.40**	**-0.21**
Vision Function and Body Performance	-0.14	-0.14	0.06	**-0.25**	**-0.02**
Social Activity	-0.75	-0.75	0.07	**-0.89**	**-0.60**

Bold font indicates a significant model.

We compared myopic and non-myopic participants (**Table 1**). No significant differences were observed between participants with and without myopia in terms of age, ethnicity, BMI, family condition, home address, or family type (*P* > 0.05). Female participants, non-alcohol consumers, non-smokers, and non-only-children had a significantly higher prevalence of myopia compared to their male counterparts, alcohol consumers, smokers, and only-children, respectively (*P* < 0.05). Additionally, participants with myopia reported lower levels of both maternal (*P* = 0.02) and paternal control (*P* = 0.03). Myopic participants also reported significantly lower SDS scores (41.26 ± 8.45) than non-myopic peers (42.55 ± 8.41) (**Table 2**).

## Discussion

This study found that vision-related QoL mediates the relationship between parental control and depressive symptoms in myopic college freshmen. Interestingly, and contrary to some previous literature, our sample showed that myopic students had a lower risk of depressive symptoms compared to their non-myopic peers.

In contrast to the previous studies, the present study found that students with myopia exhibited a lower risk of depressive symptoms. While previous studies have reported associations between visual impairment and negative psychosocial outcomes (46, 47), few investigations align with our findings. A community-based survey reported positive associations between myopia and depressive symptoms among older adults (24), whereas other studies found no significant association. For example, a school-based study detected no difference in depression rates between myopic and non-myopic students (13), and research during the COVID-19 pandemic reported no association among Chinese university freshmen (14). This apparent inconsistency can be explained through several mechanisms. First, sampling heterogeneity may account for the discrepancy. Myopia is commonly associated with higher educational attainment and superior academic performance. Although high-achieving students are not immune to distress, converging evidence indicates that greater educational attainment predicts a lower long-term risk of depression, while robust academic self-efficacy has been shown to protect against depressive symptoms (48). Second, a potential “parental-attention buffering effect” may operate. Myopia onset and progression are frequently accompanied by intensified parental investment, potentially increasing parent-child interaction and emotional support. Consistent with the stress-buffering model, such support could create a psychological cushion against academic stress and other adversities (49). In line with this interpretation, myopic students reported significantly higher parental-care scores than their non-myopic peers (mother: 23.21 ± 4.98 vs. 21.96 ± 5.36, *P* = 0.004; father: 20.92 ± 4.29 vs. 19.77 ± 4.75, *P* = 0.001). For first-year undergraduates navigating the transition from family to university, such support is a critical mental-health resource. Third, myopia is typically accompanied by intensive study engagement, and academic achievement itself has been linked to antidepressant factors, including elevated self-esteem, future orientation, and problem-solving capacity (50).

Our analysis further revealed that parental control may contribute to the risk of depressive symptoms through its association with reduced vision-related QoL across emotional function, vision function and body performance. Parents who exert excessive control, possibly motivated by academic anxiety, may encourage their children to engage in prolonged near-work and limit outdoor activities, a pattern that has been empirically associated with accelerated myopia progression (51, 52). As myopia progresses, adolescents may experience increasing limitations in sports participation, object recognition, and social interaction, potentially leading to a decline in QoL. Previous studies have consistently demonstrated that reduced vision-related QoL is significantly associated with elevated anxiety and depressive symptoms (53, 54). Vision impairment itself might not be the sole determinant, rather, the cascade of secondary declines in QoL, such as academic difficulties, activity restrictions, and apprehensions about future development, may constitute a critical pathway to depressive symptoms (55). Further evidence indicates that adolescents exposed to greater parental psychological control appear more susceptible to emotion-regulation deficits, diminished self-efficacy, and internalizing problems (56, 57). In other words, overcontrol might not only exacerbate global negative affect but also directly impair the “emotional function” dimension of vision-related QoL. Frustration, anxiety, and irritability resulting from visual impairment could be intensified by parental reproach and intrusive intervention. Such intrusive behaviors may compromise emotional autonomy, impede adaptive emotion regulation, and culminate in the accumulation of negative affect, thereby increasing the risk of depression (58, 59).

Several limitations should be acknowledged. First, the sample comprised predominantly college freshmen, which limits generalizability to other age or educational groups. Second, the sample was drawn from a single university. While this homogeneity strengthens internal validity for this specific population, it inherently constrains the generalizability of the findings to diverse age cohorts, educational tiers, or students from institutions with varying academic profiles or geographic contexts. Furthermore, data collection occurred during the COVID-19 pandemic, a period of heightened psychological stress globally (60, 61). The depressive symptoms we observed may therefore reflect this unique context, limiting the generalizability of the absolute prevalence of symptoms to non-pandemic periods. Therefore, extrapolating these results to broader populations or drawing nationwide inferences requires caution, the current findings are most relevant for understanding trends in populations exposed to intense academic demands and comparable myopia prevalence. Third, although we retrospectively assessed parenting styles and contemporaneously measured vision-related QoL and mental-health status, the cross-sectional design precludes the establishment of causal relationships. For instance, it remains unclear whether parental control precedes declines in vision-related QoL or whether pre-existing mental-health issues elicit stricter parental supervision. Without longitudinal tracking to document the order of variable emergence and to disentangle potential bidirectional influences, assertions about causal directions remain speculative. Thus, longitudinal data with repeated measurements are needed to clarify these relationships. Finally, parental control, vision-related QoL, and depressive symptoms were self-reported via questionnaires, rendering the data susceptible to bias. Specifically, recall bias may have distorted retrospective reports of parental control, as participants might inaccurately reconstruct past experiences. Furthermore, social-desirability bias could have influenced participants’ responses: they might have underreported parental control to align with societal norms regarding autonomous college life or downplayed depressive symptoms to avoid stigma.

## Conclusion

In this cross-sectional study of college freshmen in Yunnan Province, China, we found that vision-related QoL significantly mediated the association between parental control and depressive symptoms, suggesting a potential mechanism for depressive symptoms in myopic adolescents. These findings underscore the importance of cultivating a supportive family environment and highlight the value of promoting autonomy-supportive parenting as part of myopia management programs to bolster adolescent mental health. Future longitudinal research is needed to confirm these causal pathways and to develop family-based interventions that can improve both visual and psychological outcomes for adolescents with myopia.

## Data Availability

The raw data supporting the conclusions of this article will be made available by the authors, without undue reservation.
