# Supplementary material for: Parental control and depressive symptoms in college freshmen with myopia: the mediating role of vision-related quality of life
Source: Front Psychiatry. 2025 Dec 26;16:1642896. doi: 10.3389/fpsyt.2025.1642896 (PMC12785179; doi:10.3389/fpsyt.2025.1642896)
Supplement: Supplementary file 1 [file SupplementaryFile1.docx]

**Supplementary Figure 1. E-values of the total effect model.**


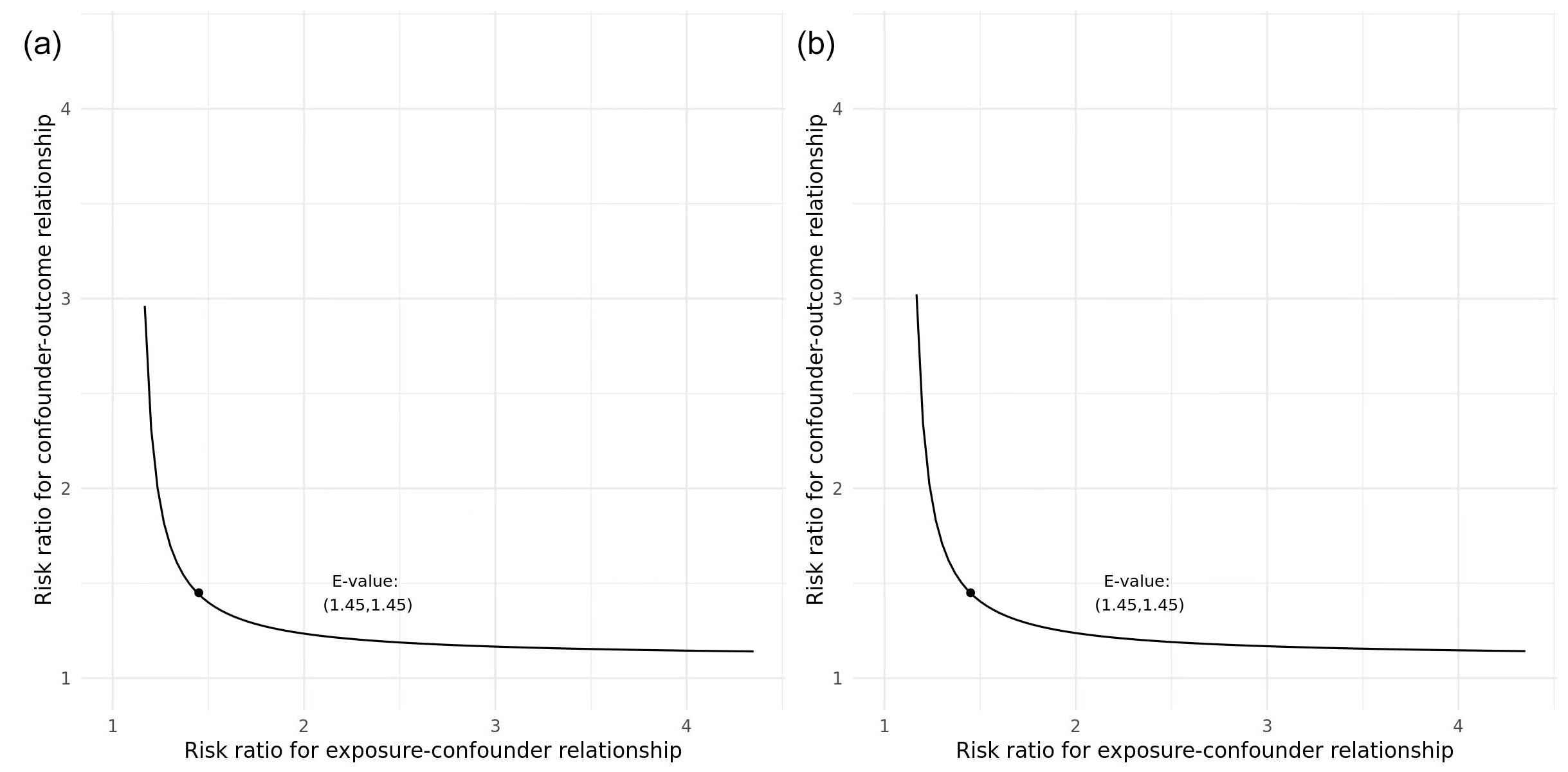
(a) Maternal control as independent variable; (b) Paternal control as independent variable.
